# Exploring Environment-Intervention Fit: A Study of a Work Environment Intervention Program for the Care Sector

**DOI:** 10.1155/2015/272347

**Published:** 2015-08-25

**Authors:** Louise Hardman Smith, Birgit Aust, Mari-Ann Flyvholm

**Affiliations:** The National Research Centre for the Working Environment, Lersø Parkallé 105, 2100 Copenhagen, Denmark

## Abstract

Targeting occupational health and safety interventions to different groups of employees and sectors is important. The aim of this study was to explore the environment-intervention fit of a Danish psychosocial work environment intervention program for the residential and home care sector. Focus group interviews with employees and interviews with mangers were conducted at 12 selected workplaces and a questionnaire survey was conducted with managers at all 115 workplaces. The interventions enhanced the probability of employees experiencing more “good” work days, where they could make a difference to the lives of clients. The interventions may therefore be characterized as culturally *compelling* and having a good fit with the immediate work environment of employees. The interventions furthermore seemed to fit well with the wider organizational environment and with recent changes in the societal and economic context of workplaces. However, some workplaces had difficulties with involving all employees and adapting the interventions to the organization of work. The findings suggest that flexibility and a variety of strategies to involve all employees are important aspects, if interventions are to fit well with the care sector. The focus on employees' conceptualization of a “good” work day may be useful for intervention research in other sectors.

## 1. Introduction

In occupational health and safety research, the importance of developing intervention programs to target different groups of employees and sectors has been emphasized in recent years [1, 2]. The WHO Healthy Workplace Framework and Model suggests that to be successful “the specific needs and requirements of the local culture and conditions should be incorporated into the health and safety activities in the workplace” [3]. Furthermore, a reconciliation of the competing paradigms of maximizing implementation fidelity versus adapting programs to fit local cultures has been proposed, because the tailoring of programs to local cultures has increasingly been recognized as an essential element for implementation. The adaption of programs should therefore receive the same attention as fidelity to the implementation of intervention activities [4, 5]. As a consequence of the recognition of the need for design of tailored interventions, a search for pathways to target interventions to specific groups of employees and workplaces such as for instance small enterprises has been intensified [6, 7].

A growing amount of scientific work has furthermore been devoted to strengthening organizational interventions, and deepening the understanding of the processes and contextual issues, that influence the implementation and outcome of interventions, for instance on employee stress and wellbeing [8]. Research on the “healthiness” of organizational change processes within this field of research has emphasized the importance of focusing on how interventions integrate with employees' experiences of work to shape their reactions to interventions. Among the factors identified as being of special interest for managing healthy organizational change are: role clarification, manager availability, attention to diversity in perceptions and reactions, the use of constructive conflicts and attention to the local norms among employees [9, 10].

Randall and Nielsen, who for years have worked on how to develop, implement, and evaluate organizational interventions for stress and wellbeing, have introduced the concept of “intervention fit” [11]. In their concept, the degree of “fit” between an occupational health and safety intervention and the specific context is viewed as determining the intervention process, and as a consequence the intervention outcomes. Interventions may in some situations be appropriate and powerful due to a good fit with the organizational context, while the same intervention might lead to weak effects in other organizations due to poor fit [11]. Randall and Nielsen recommend that at least four levels of environment-intervention fit should be addressed in evaluations of organizational interventions. The levels are as follows: the individual employees' immediate working environment, the environment of the team they work in, the wider organizational environment, and the societal and economic context of the organization.

Based on a social ecological theoretical perspective, Panter-Brick et al. [12] have taken the idea of fit between an intervention and the context, in which the intervention is planned to be implemented, a step further. They advocate that health interventions to be successful should “strive to be culturally* compelling*, not merely culturally appropriate”. They argue that to be culturally compelling, health interventions should build on “existing practices, skills and priorities”. Culturally appropriate interventions are defined by Panter-Brick et al. as interventions that take into account psychosocial variables such as attitudes, norms, and self-efficacy, which may determine intention to change. Culturally* compelling* interventions, however, extend wider due to their focus on how to propel “intention to change” to “actual behavior change.” Panter-Brick et al. propose that key determinants of culturally compelling interventions are related to the choice of trigger, the sense of community ownership, and the fit of interventions with local priorities [12].

The aim of the current mainly qualitative study was to explore the environment-intervention fit of a sector-specific psychosocial intervention program for the home and residential care sector (care sector in the following). Inspired by Randall and Nielsen, we investigated different levels of environment-intervention fit. However, because work in the care sector to a large extend is organized in teams, we did not distinguish between the individual employees' immediate working environment and the environment of the team they work in. Instead, we investigated the following three levels of fit: (1) the fit of the interventions with employees' and their teams' immediate working environment combined, (2) the fit of the interventions with the wider organizational environment, and (3) the fit of the interventions with the societal and economic context of workplaces.

Furthermore, the analytical approach of the study was inspired by the concept of “culturally* compelling*” interventions introduced by Panter-Brick et al. [12]. Based on this approach, we propose that one way for interventions to be culturally compelling is by enhancing the probability of employees experiencing more of what they conceptualize as a “good” work day. This approach focuses on how to enhance the positive aspect of work seen from employees' perspectives, which is in contrast to much work environment intervention research which primarily focuses on reducing shortcomings and problems in the work environment.

The intervention program for the care sector evaluated in this study was developed in connection with a Danish government-financed occupational health and safety initiative for sectors threatened by attrition. The interventions were called “prevention packages” and The Danish National Research Centre for the Working Environment evaluated the interventions at the workplaces that had applied for an intervention the first year after the launch [6].

## 2. Material and Methods

The intervention program for the care sector (homecare, nursing homes, and institutions for the physically and mentally disabled) included four different prevention package interventions. The interventions were primarily designed to improve psychosocial wellbeing, but physical work environment issues could also be addressed if the employees chose so.

The specific focus of the interventions differed, but all of the interventions included participatory processes where employees themselves identified and found solutions for the problems they experienced in their everyday work. Such participatory processes have been related to responsibility for and commitment to changes, as well as improvement of health and safety at workplaces [13–15].

The four interventions for the care sector were as follows.

1. *Peer Coaching.* In this intervention employees were introduced to a method to give and receive guidance and support to and from colleagues in relation to difficult work situations with clients. As part of the intervention a municipal consultant educated four key employees to become trainers for peer coaching sessions. After the training the key employees conducted sessions of peer coaching for colleagues by themselves.
2. *The Three Most Resource-Demanding Clients.* This intervention focused on teaching employees a method to engage in structured collegial dialogue. The method guided the employees to find new ways to solve problems related to the three most resource-demanding clients. The nine key employees who worked the most with these three clients participated in working groups.
3. *Future Workshop*. This intervention consisted of a workshop for all participating employees, guided by a municipal consultant. A future workshop is an established method for developing improvement ideas. The workshop included a critical reflection regarding daily routines, development of visions and ideas for improvements and finally the formulation of a realistic action plan [16]. After the future workshop a number of specific improvements were implemented.
4. *Occupational Health Circle*. Health circles consisted of managers and a group of employees representing all employees with regard to different tasks, seniority, and so forth. Based on an assessment of the work environment and during a course of regularly scheduled work meetings led by the municipal consultant, the group developed appropriate suggestions for improvement that were then discussed with the entire group of employees [13, 17]. Eight to ten employees and management representatives participated in the health circle.

Depending on which of the four interventions was chosen, the duration of the intervention was between six and nine months. Financial compensation for salaries was given for between 28 and 40 working hours for key employees, and somewhat less for managers and other participating employees. Compensation for municipal process consultants was given for between 74 and 106 hours of support by consultants.

In the care sector 115 workplaces were granted a total of 133 prevention package interventions the first year after the launch (some workplaces had applied for and were granted more than one prevention package). Based on the workplace applications it was calculated that a total of 4,330 employees would participate in the interventions.

### 2.1. Analytical Approach

We investigated how the prevention package interventions for the care sector fitted with level one, the employees' and their teams' immediate working environment, by relating employees' perceptions of the impact of the interventions to their conceptualizations of a “good” work day. Our proposition was that if employees' perceptions of the impact of the interventions seemed to enhance the probability of them experiencing more “good” work days, the interventions could be characterized as being culturally* compelling*, and the intervention fit with employees' immediate working environment would be interpreted as a good fit. To determine how the interventions fitted with level two, the wider organizational environment, we investigated whether the interventions seemed to support the main tasks of the workplaces and could be adapted to the everyday organization of work. The fit of the interventions with level three, the societal and economic context of workplaces, was determined by investigating whether the interventions seemed to match the changes in the societal and economic context the participating workplaces had experienced the year before the intervention. Because the interventions were mostly aimed at changes in employees' immediate work tasks and environment, most emphasis was put on exploring level one: the intervention fit with employees' and their teams' immediate working environment.

### 2.2. Data Collection

#### 2.2.1. Interviews and Observations

For each of the four interventions for the care sector, three workplaces were chosen from different geographical areas and from both home and residential care. At these 12 workplaces semistructured focus group interviews with employees, individual interviews with managers [18–20] and observations of staff meetings were performed. The focus group interviews and the interviews with managers were conducted in connection with the termination of the interventions. Four to 10 employees representing the employees at a particular workplace were interviewed in focus groups at each of the 12 selected workplaces [20]. The employees who participated in the focus groups were selected by their managers. They included both employees who had key roles during the interventions, such as being a member of the steering committee and participant in a “health circle” or in a working group in “the three most resource-demanding clients,” and employees who had not performed such roles. The employees were informed by their managers that participation in the focus groups was voluntary. Additionally, the researchers in charge of conducting the interviews informed all interview persons that participation in the interviews was voluntary and that confidentiality was guaranteed. According to Danish National Committee on Biomedical Research Ethics (http://www.cvk.sum.dk/), ethical approval in Denmark only applies for studies using biological material.

In the beginning of the focus group interviews, we asked employees to discuss and describe a “good” work day in detail in their own words. Thereafter we asked employees to describe and discuss experiences with the interventions and their perceptions of the impacts of the interventions. Employee perceptions with regard to these two main topics informed the exploration of the intervention fit with employees' and their teams' immediate working environment. The focus group interviews lasted between one hour and one and a half hours. Examples of questions asked in the focus group interviews are presented in Table 1.

Interviews with managers focused on their experiences with the implementation and outcome of the interventions. Most of the interviews lasted about half an hour; however one third of the interviews lasted between half an hour and one hour. Examples of the questions asked in the interviews are presented in Table 1.

Observations were conducted during staff meetings at mid-term and the end of the intervention, where employees were informed about the status of the intervention. The observations of staff meetings included counting how many employees were present and observations of how employees conducted or presented intervention activities. We primarily focused on how many employees seemed to be informed and interested in the activities as well as the character of the verbal and the nonverbal communication between employees and between employees and managers (e.g., the level of detail by which activities were presented and discussed).

Three-quarters of the interviews and observations were conducted by the first author who has considerable experience in qualitative research interviewing [21, 22]. The rest of the interviews and observations were conducted by research assistants under the guidance of the first author. All interviews were audio-taped, transcribed verbatim by research assistants, and coded by the first author in accordance with the topics of the interview guide using the software program Nvivo 9. Based on the coded interview material, thematic analysis within the analytical framework of the study was conducted, and themes that best represented the entire data set were derived from an experience based and creative analytical process [23]. The analytical process included reviewing the themes, defining and naming themes, and selecting extract examples from the data set, besides a final analysis relating back to the research questions and the theoretical and analytical framework.

#### 2.2.2. Questionnaires

Self-administered questionnaires were sent to managers at the beginning and at the end of the interventions at all workplaces conducting one or more of the four interventions the first year after the launch. Of the 133 managers who participated, 121 answered the questionnaire at the beginning (91%) and 105 at the end (79%). Questionnaires to managers at the beginning of the interventions included questions about a range of selected changes in the societal and economic context of the workplace such as budget and staff cuts the year before the intervention.

The questionnaires to managers at the end of the interventions included items about the adaptability of the interventions. This questionnaire also included optional open-ended questions about experiences with the interventions and the implementation process, which 91 managers chose to answer. The questionnaires at the end furthermore included open-ended questions about suggestions for improvements of the interventions, which 65 managers answered. The items in the questionnaire were primarily chosen among items which had previously been used in evaluations by The National Research Center for Work Environment, but some were developed specifically for this study. The open-ended questions were similar to the overarching questions asked in the interviews with managers (Table 1).

The questionnaire survey and the interviews were conducted separately. However, the final questionnaire survey to managers and the interviews with managers both took place in connection with the termination of the intervention. Some of the managers had answered the questionnaire at the end of intervention before the individual interview, while others answered it after the interview, depending on when the questionnaire was sent to them and when the interview was conducted.

We compared and analyzed the answers to the open-ended questions in the questionnaire in combination with the material from the interviews with the managers to determine how the interventions seemed to fit with the main tasks and organization of work at the workplaces. The answers to the open questions in the questionnaire were categorized, in a summative analytical process, according to how many managers expressed the same experiences and suggestions i.e. many, several or some managers.

## 3. Results

### 3.1. The Fit of the Interventions with Employees' and Their Teams' Immediate Working Environment

#### 3.1.1. Employees' Descriptions of a Good Work Day

In the analysis of the focus group interviews we identified five distinct themes regarding the employees' descriptions of a “good” work day. The themes and excerpts from the interviews illustrating the themes are presented as follows.

*(1) “Make a Difference” for the Clients.* Employees described that having good contact with clients and being able to give meaningful care to clients were part of a good work day for them. They described situations where they could “make a difference” to the lives of clients for example, by providing better treatment, and thereby help to improve the clients wellbeing or heath status.

*“It is a day where you have been in contact with each resident and heard what they are preoccupied with or have given them an offer of activity” (workplace 3).*

*“It can be when you refer someone to physiotherapy and it goes through, and it makes a difference to the client, or it can be when you identify that a client with mental illness is becoming worse and you can make sure he or she gets the necessary treatment” (workplace 11). *

*(2) Sufficient Resources to Do a Good Job.* This theme was related to the availability of sufficient resources, so the employees could do what they were supposed to do in a day's work and in a quality they were satisfied with. The resources primarily referred to were economic resources and the number of care personnel. High absenteeism and widespread use of temporary workers was especially highlighted as a problem. A good work day was described as a day with low absenteeism and availability of all necessary resources so tasks could be conducted as they were supposed to be.

*“There are not too many colleagues sick. We are the number of employees we should be and are able to perform our services” (workplace 3).*

*“You get done what you need to do and resolve tasks in a way that you are satisfied with” (workplace 6).*

*(3) Good Social Relationships with Colleagues.* This theme involved the possibility of interaction with colleagues in a respectful and friendly manner. Respectful interaction referred to the recognition of skills of each other and specific areas of knowledge such as care for clients with dementia or lifting of clients. Friendly interaction referred to reciprocal interest and care for each other also with respect to personal problems

*“It is when there is a good tone between us, and we have respect for each other, both professionally and personally” (workplace 1).*

*“It is if we also have time for some small talk for instance if a colleague is upset” (workplace 3).*

*(4) Good Collaboration*. This theme involved having professional collaboration and the possibility of discussing work with colleagues, so the care for clients could be optimized.

*“A good work day is when I feel we are a team, where observations of clients and health initiatives merge” (workplace 3).*

*“We have the possibility of discussing work situations with colleagues and experiencing a positive development with a client due to this” (workplace 9).*

*(5) Good Management.* This theme referred to the importance of visible leadership and good planning of work tasks by managers, so the care for clients was not disrupted by disputes or lack of knowledge about how to solve work tasks or who was responsible.

*“A good work day requires good planning from management” (workplace 2).*

*“A good work day requires visible leadership and clear guidelines” (workplace 7).*

In summary, all themes identified in employees' conceptualization of a “good” work day contributed to making a difference to the lives of clients. The other themes could be interpreted as subordinate but important preconditions to fulfill this overarching aim: Good social relationships with colleagues are a precondition for good collaboration; good management and sufficient resources are preconditions for performing a good job, which consisted of making a difference to the lives of clients.

#### 3.1.2. Employees' Perceptions of the Impact of the Interventions

The analysis of the focus group interviews showed that a large majority of the employees perceived, that the interventions had contributed to positive changes in their wellbeing and work environment. However, some employees, primarily among those who had not had key roles during the interventions, perceived that the interventions had only had minor or no impact. As was the case for the description of a “good” work day, we identified five distinct themes in employees' perceptions of the impact of the interventions. The themes and excerpts from the interviews illustrating the themes are presented as follows.

*(1) Improved Possibility for Reflection and Discussion of Work with Colleagues.* The employees in the focus groups expressed that the interventions had given them time for reflection and improved possibility of discussing difficult and straining work situations. Furthermore, employees reported that the discussions had resulted in a broader variety of viewpoints on how to carry out a work task and how to solve a problem.

*“The project has given us the possibility to talk about the things that are straining in our work” (workplace 1).*

*“We have had time to work thoroughly with some of our challenges in regard to clients. Usually there is not time for this, although there ought to be” (workplace 6).*

*(2) Improved Communication.* Participants pointed out that the interventions had entailed clearer communication internally in the group of colleagues as well as an improved tone. Furthermore, employees emphasized that they listened to one another with more attention.

*“People listened to each other in a different way compared to how we usually discuss problems at staff meetings” (workplace 2).*

* “The tone at the workplace has become more comfortable. We have also become clearer in our communication” (workplace 11).*

*(3) Improved Interaction and Knowledge of Colleagues' Perspectives.* Another aspect described by the participants of the focus group interviews was that the interventions, through the increased number of meetings and activities across employee subgroups, led to more social interaction with colleagues. Furthermore, intervention activities in smaller groups had given those, who usually did not make themselves heard at large group meetings, the opportunity to express their viewpoints.

*“It has been an opportunity to see another side of ones colleagues, which is positive” (workplace 3).*

*“Those who do not usually say much have expressed their views much more” (workplace 7).*

*(4) Improved Methods to Work Systematically with Work Tasks and the Working Environment.* Participants of the focus group interviews pointed out that the methods introduced to them, during the intervention, had helped them to work more systematically with how to solve their work tasks, and how to improve their working environment. The specific methods described by the employees depended on which of the four interventions had been carried out at their particular workplace.

*“The coaching has given us the possibility to talk about the most straining aspects of our work” (“peer coaching”, workplace 1).*

*“I think it is really important that everyone involved in this project has a shared understanding. It has been interesting to examine a client's background. It gives an understanding of why he or she acts the way they do” (“the three most resource-demanding clients”, workplace 5).*

*“We have become more aware of each other's resources. We have experienced more influence on our work through the project. We have also become more creative” (“future workshop”, workplace 8).*

*“We have worked constructively in the group. We have learned to work in a completely different way than before” (“occupational health circle”, workplace 12).*

*(5) Minor or No Impact of the Interventions.* Some participants of the focus groups perceived that the interventions had only contributed to minor or no changes. They, for instance, did not find the interventions relevant or sufficient to address the major problems in their work environment, or assessed the fact that the intervention had taken time away from important everyday work tasks.

*“I do not think it has made a difference. We have much greater problems in our psychosocial work environment than the small problems that have been dealt with” (workplace 11).*

*“I thought ‘what is it good for'? We are dissatisfied because we do not have sufficient time. I am responsible for the medicine and I have not had time to learn the computer system. Meetings are not always helpful in my opinion” (workplace 12).*

To sum up, the themes in employees' perceptions of the impact of the interventions highlight that the interventions had provided time to reflect on work and discuss potential new approaches with colleagues. More social interaction in connection with small team meetings as well as meetings across teams had improved communication and allowed a larger variety of different perspectives, including inputs from those who typically did not express their opinion as much. In addition, the interventions supplied employees with different approaches on how to discuss work in a systematic manner and implement new methods which had improved collaboration. The analyses, however, also showed that some employees perceived that the interventions had not had any impact or had even had a negative impact on everyday work, by taking time away from more important tasks.

When we compared employees' descriptions of a “good” work day with their perceptions of the impact of the interventions, we found that, according to the large majority of employees, the interventions improved the possibilities for reflection and discussion of work situations with colleagues. In addition, the interventions entailed improved methods to work systematically with work tasks. Furthermore, the interventions improved communication and interaction with colleagues which gave the possibility of establishing good social relationships, which are a precondition for good collaboration. Combined, these improvements contributed to the possibility of doing a good job and making a difference to the lives of clients, which was the main intention in the employees' descriptions of a “good” work day. However, according to the employees the interventions had primarily influenced relations between colleagues, whereas nothing was reported about the influence on management, which was also an aspect of a “good” work day. With this exception, the impacts of the interventions seem to enhance the probability of experiencing more of what employees conceptualized as a “good” work day. Based on this, we characterize the interventions as being culturally compelling, and conclude that there was a good fit of the intervention and the immediate working environment of employees and their teams.

### 3.2. The Fit of the Interventions with the Wider Organizational Environment

The questionnaire survey showed that 85% of the managers reported that the intervention they had chosen could be adapted to their workplace. This result was supported by the open-ended answers in the questionnaire and in the interviews. However, these data sources also gave an insight into the difficulties the workplaces had experienced with the adaption of the interventions.

*Most of the managers* reported satisfaction with the focus areas of the interventions in answers to the open-ended questions in the questionnaire at the end of the interventions and in the interviews.* Most of the managers'* furthermore expressed that the interventions had contributed to a greater degree of professionalism at their workplace and that communication and cooperation between employees had improved.* Several managers* emphasized that a greater sense of social community among employees had been established and that communication and cooperation with clients and their relatives had improved. According to these managers the interventions had contributed to improving the main tasks related to the client-related work at their workplace. All of these experiences pointed at a good fit between interventions and the wider organizational environment.

*Some managers*, however, reported that they had experienced challenges in connection with the adaption of the interventions to the everyday organization of work. These managers recommended a higher degree of flexibility in the interventions. They would have preferred to be able to plan the implementation process so that it would fit better to their particular workplace. Some, for example, would have liked to have had the possibility of determining the length of meetings, number of key employees, or the number of clients who should be involved in the intervention.

According to some of the managers it was furthermore a challenge to inform, involve, and engage the employees who were not the primary participants in the interventions such as key personnel or members of working groups. In all interventions for the home and residential care sector, information about the intervention process was delivered during meetings at start-up, mid-term, and at the end of the interventions, where all employees were supposed to be present. The observations of the meetings, however, revealed that far from all of the participating employees were present at the meetings. Furthermore, some of those who were present knew little about the progress of the intervention, because they had not participated in the previous meeting. When this observation was presented in the interviews with managers, some explained that joint staff meetings were rarely held because employees worked in shifts. Moreover, if joint meetings were held they were usually voluntary for employees who were not working on the day of the meeting. Managers explained also that even when there were written records of the meetings or the progress was depicted in other ways such as on posters or boards, they were not always read by employees.* Some managers* would therefore have liked to have had the possibility of conducting more of the smaller team meetings instead of the scheduled large joint meetings where all employees were supposed to be present.

### 3.3. The Fit of the Interventions with the Societal and Economic Context of Workplaces

*Most mangers* reported satisfaction with the financial compensation for time spent on the interventions and the consultancy support to implement the interventions. This support fitted well with the societal and economic context of workplaces in the care sector, because it counter-balanced a context where the majority of managers reported that they had experienced major changes in the year before the intervention: 53% of the managers reported budget cuts and 60% reported reduction in number of employees (Table 2).* Several managers* reported that the prevention package interventions had been welcomed, because the budget cuts and the many other changes that had occurred in recent years, had not left much time or resources to work specifically with the working environment.

Based on the experiences of the managers, we conclude that the interventions seemed to fit well with the societal and economic context of workplaces.

## 4. Discussion

A large majority of employees' participating in the focus groups perceived that the interventions had contributed to positive changes in their working environment. According to these employees the interventions had improved the possibilities for reflection and discussion of work situations. Furthermore, better communication and collaboration as well as improved methods to work systematically with work tasks were emphasized. The employees' perceptions of the outcome of the interventions were supported by the managers who reported that the interventions had contributed to improve collaboration and the main tasks related to the client-related work at their workplace.

In their description of a “good” work day, employees emphasized the importance of being able to perform work of a good quality and make a difference to the lives of their clients. In addition, they stressed possibilities to collaborate and interact with colleagues as well as the importance of good management. Thus, when perceptions of the impact of the interventions were related to descriptions of a “good” work day, it appeared that the changes achieved by the interventions seemed to enhance the probability of employees experiencing more “good” work days. The interventions can therefore be characterized as culturally compelling and fitting well with the immediate work environment of employees and their teams. However, the interventions did not seem to have contributed to the management of work, which also was pointed out as one aspect of a “good” work day. This may be explained by the fact that management had not been a specific focus of the interventions.

We are not aware of other studies that have focused specifically on the conceptualizations of a “good” work day among employees in the home and residential care sector. However, several studies confirm selected aspects of our findings. For example, studies have found that the direct care relation with the elderly is associated with the experience of meaning of work [24–27] and that the possibility of adapting care to the individual and the specific work situation are crucial for the perception of conducting satisfactory work in health care [26]. Furthermore, it has been shown that improvements in time available for the contact with the elderly, increased skill discretion, and improved social relations could prompt previous eldercare employees to reconsider quitting or retiring [28].

Our study also showed that some employees did not perceive that the intervention had resulted in any improvements. Some of these employees reported that the interventions could not improve employee wellbeing or solve the main problems, their workplace experienced in the psychosocial work environment. In a context of major reductions in resources and number of employees, the prevention package interventions offered might not have been sufficient to address all potential psychosocial strains and that is why more extensive measures may be needed in some cases.

A large majority of the managers reported that the interventions could be adapted to their workplace, and they were satisfied with the focus areas of the interventions, which they perceived fitted well to the main tasks related to the client-related work in the sector. This points at a good intervention fit with the wider organizational environment. However, the division of employees with key roles and more peripheral participants in the interventions received some criticism, because it had been difficult to inform, involve, and engage employees without active roles in the intervention activities. Moreover, for some of the workplaces, there was a mismatch between the intervention setup and the meeting practices related to shift work.

The managers revealed that financial compensation for time spent on the intervention, and consultancy support to implement the interventions, fitted well with the recent changes in the societal and economic context of workplaces in the care sector. Savings in public spending partly due to the financial crisis in recent years had been a main problem in the eyes of many managers.

Several managers recommended that the interventions should be more flexible, if they should be able to fit with the daily organization of work. While more flexibility might improve implementation, too much flexibility can jeopardize the implementation fidelity needed to achieve a desired impact, as mentioned in the introduction [4, 5]. In our case the prevention package interventions were funded by public means. This contributed to a more restrictive approach in the guidelines for the first interventions focusing largely on implementation fidelity. Based on the experiences with these interventions, the interventions developed since have become more flexible allowing a greater degree of adaption to local workplace cultures and contexts, including, for example, more flexible meeting structure for workplaces with shift work.

The results of this study may provide inspiration for future targeted occupational health and safety interventions in the care sector or similar sectors. If interventions in the sector intend to support the positive aspects of work seen from the insider's perspective, they should enhance the possibility for employees to make a difference to the lives of their clients and to perform work of good quality, or at least not be in conflict with these aims. Interventions aiming to support what employees in the care sector define as a good work day should also ensure the possibility to interact and collaborate with colleagues and to focus on a good management of work tasks. Furthermore, our study shows that strategies to involve all employees should be developed further and flexible concepts should be introduced, so interventions can be adjusted to the specific workplace context. Based on our findings, we propose that to design workplace interventions that will fit with the targeted groups of employees and their environment extensive workplace visits and thorough reflections on how to make an intervention fit with conceptualizations of a “good” work day and the everyday organization of work are recommended.

The conceptual focus on the importance of intervention fit with employee conceptualizations of a “good” work day for intervention success may serve as inspiration for intervention research in other sectors. The focus is to some degree aligned with and may therefore be of interest to the field of research that is engaged in “healthy” management of change processes. This research focuses on how interventions integrate with employees' experiences of work to shape their reactions to the interventions [9, 10]. Furthermore, the fit of an intervention with employees conceptualizations of a “good” work day may provide a supplement to the common variables that have been linked to employees appraisal of interventions, such as their influence over intervention content, the quality of an intervention, and its sustainability [29].

### 4.1. Strength and Limitations of the Study

The strength of the study is the detailed investigation of the intervention-environment fit of the implemented interventions with a specific focus on employees' perceptions. By exploring what employees consider as a “good” work day and comparing these aspects with their perception of the impact of the interventions, we were able to investigate to what extend the interventions were culturally compelling, that is, achieving a good fit with the employees and their teams immediate work environment. The study thereby contributes to a better understanding of the mechanisms that can support or impede the implementation of work place interventions.

A limitation of the study is that the participants in the focus groups were selected by the managers. Even though we asked the participants to reflect on the viewpoints of their nonparticipating colleagues, the participants may have rated the interventions more positive than their colleagues. Furthermore, a limitation was that focus group interviews with employees and the questionnaire survey and interviews with managers were conducted in connection with the termination of the interventions. A later follow-up could have investigated if the perceived positive impacts of the interventions were sustainable, and if positive impacts were disseminated to employees without key roles in the interventions.

## 5. Conclusions

Results from the present study may provide inspiration for the design of future targeted interventions which aim to improve work environment and employee wellbeing in the care sector. To achieve a fit between the immediate work environment and employees' conceptualizations of a “good” work day, the interventions should enhance the possibility for employees to make a difference to the lives of their clients, to perform work of good quality and interact and collaborate with colleagues and to contribute to good management. Furthermore, to fit with the wider organizational environment as well as societal and economic context, strategies to involve all employees should be considered and flexible concepts should be introduced, so interventions can be adjusted to the specific context of the workplace. The focus on employee conceptualizations of a “good” work day in this study may be useful for intervention research in other sectors, which aims to design culturally compelling interventions.

## Figures and Tables

**Table 1:** Topics in the interviews with employees and managers.

Topics in the focus group interviews with employees	Examples of questions
Perceptions of a good workday	(1) Describe a good work day: What happens during a good work day from start of shift to end of shift? What does a good workday look like in regard to, for example, main tasks and interaction with colleagues? (2) Further questions and probes about a good workday: Could you give some examples? What happens more? Do you others agree? Do you think colleagues, who are not participating in this focus group, have other perceptions? If so, which? Does anyone have other examples?

Perceptions of the impacts of the interventions	(1) Has the intervention had any impacts? If so which impacts? Has the daily work changed? If so how? What has been the most significant change? What has been the best thing about the intervention? What has been annoying, not so good, or deteriorated due to the intervention? (2) Further questions and probes about impacts of the intervention: Could you give some examples? What has happened more? Do you others agree? Do you think colleagues, who are not participating in this focus group, have other perceptions? If so, which? Does anyone have other examples? (3) Would you recommend the intervention to other workplaces similar to yours? If so, what would you tell them they could achieve from it?

Additional comments	Do you have any other comments about the intervention?

Topics in the interviews with managers	

Perceptions of the implementation of the interventions	(1) Was it possible to adapt the intervention to your workplace? Was the time schedule realistic? Have you been able to implement all the activities? Was the financial support sufficient? Have you changed the intervention so it could be adapted better, and if so how? What did not work well and why? (2) How could the intervention be improved? (3) What has been the best and the most challenging part of the project?

Perceptions of the impacts of the interventions	(1) Has the intervention had any impacts? If so, which impacts? Has the intervention changed any work routines, if so, which and how? What have been the most significant changes? What impact has the intervention had all in all? (2) Has the intervention met your expectations? (3) Would you recommend the intervention to other workplaces similar to yours? If so, what would you tell them they could achieve from it?

Additional comments	Do you have any other comments about the intervention?

**Table 2:** Managers' reports of changes at the workplace the year before the intervention.

	Changes the year before the intervention	
	% answering “yes”	Number of respondents
Budget cuts	53	114
Reduction in the number of employees	60	117
Restructuring (e.g., mergers)	49	113
New tasks	64	114
Changes in management	31	114
